# A Retrospective Study of Infant and Maternal Risk Factors in LUMBAR Syndrome

**DOI:** 10.1002/mgg3.70093

**Published:** 2025-04-07

**Authors:** Denise W. Metry, Dawn H. Siegel, Kim M. Keppler‐Noreuil

**Affiliations:** ^1^ Department of Dermatology Driscoll Children's Hospital Corpus Christi Texas USA; ^2^ Department of Dermatology, and by Courtesy, Pediatrics, School of Medicine Stanford University Palo Alto California USA; ^3^ Department of Pediatrics, Division of Genetics and Metabolism University of Wisconsin School of Medicine and Public Health Madison Wisconsin USA

**Keywords:** demographics, LUMBAR syndrome, OEIS complex, PHACE syndrome, risk factors, segmental hemangioma, Septo‐optic dysplasia

## Abstract

**Background:**

LUMBAR syndrome is the association of segmental infantile hemangiomas that affect the Lower part of the body with Urogenital anomalies, hemangioma Ulceration, spinal cord Malformations, Bony deformities, Anorectal malformations, Arterial anomalies and/or Renal anomalies. The etiology is not known but is suspected to be multifactorial, involving genetic and environmental factors.

**Methods:**

We retrospectively reviewed a large database of 109 published reports of LUMBAR syndrome to study potential associated clinical risk factors, the first such effort.

**Results:**

LUMBAR is significantly more common in full‐term, normal birth weight, singleton girls. We found no statistically significant differences in disease severity between affected girls and boys. There were no reports in twins or other multiple births, no reports of familial recurrence, and no repeated maternal illnesses, exposures, or other prenatal risk factors.

**Conclusions:**

Prospective studies in LUMBAR syndrome are needed to further evaluate maternal risk factors for prenatal hypoxia, gene–environment interactions, and genetic susceptibility variants.

## Graphical abstract

A database of 109 published individuals with LUMBAR syndrome was used to identify potential infant and maternal risk factors. Results showed that LUMBAR syndrome is significantly more common in full‐term, normal birth weight, singleton, girls. There were no reports in twins or other multiple births, no reports of familial recurrence, and no repeated maternal illnesses, exposures, or other prenatal risk factors.

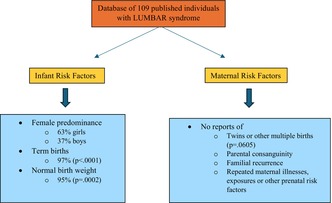

## 1 Introduction

LUMBAR syndrome is an acronym for Lower body hemangiomas, Urogenital anomalies, and hemangioma Ulceration, spinal cord Malformations, Bony deformities, Anorectal/Arterial anomalies, and Renal anomalies. The syndrome is rare, with approximately 150 published reports to date, and of unknown cause. Diagnostic criteria for LUMBAR were recently established via Delphi consensus of an international panel of pediatric specialists. The diagnosis requires the presence of a characteristic segmental infantile hemangioma (IH) of the skin and one other regional, extracutaneous congenital anomaly affecting the lower body anatomy (Iacobas et al. 2010; Metry et al. 2024).

**FIGURE 1:**
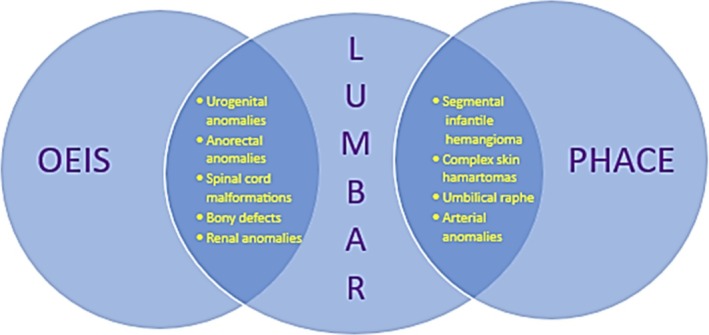
Overlapping features between LUMBAR syndrome with PHACE syndrome and OEIS complex.

**FIGURE 2:**
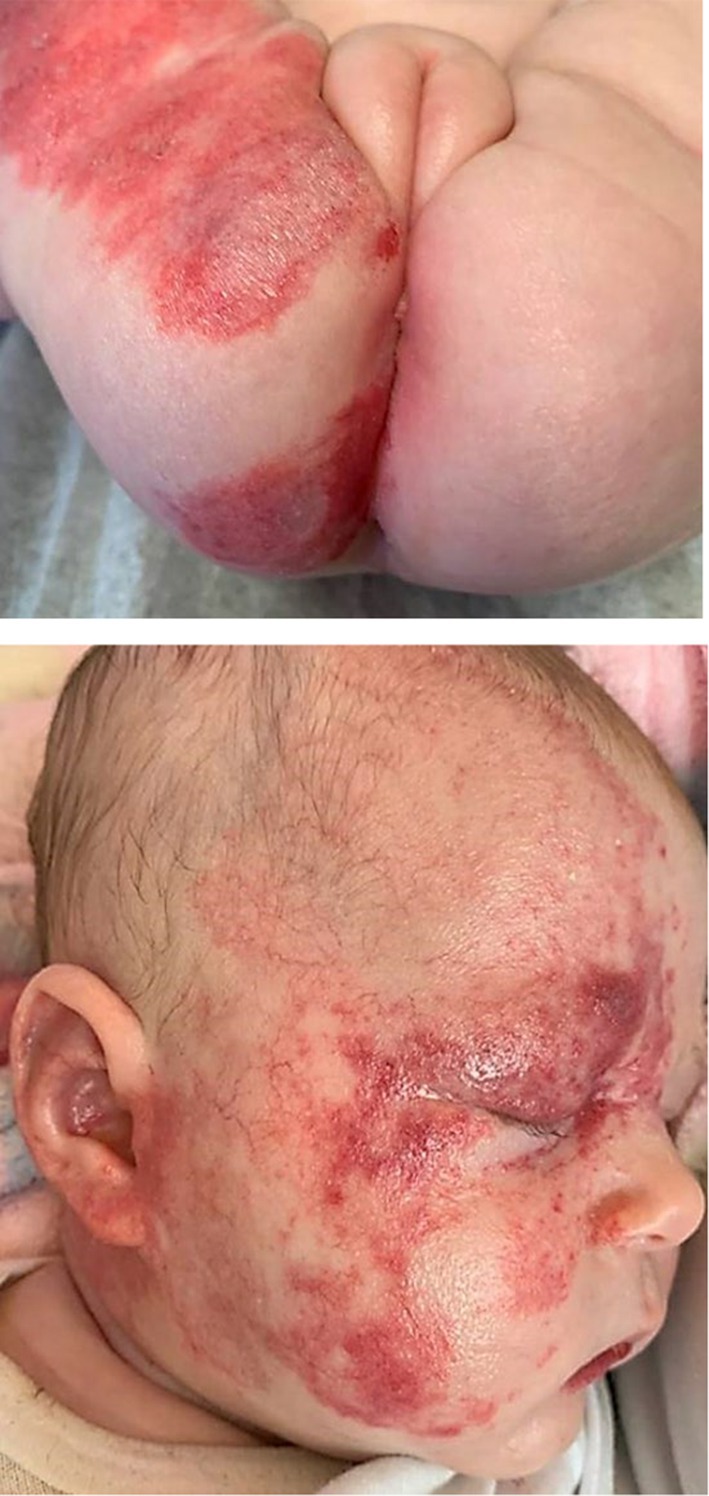
(a, b) Infant with segmental infantile hemangiomas of the face, buttocks, and pelvis at risk for both LUMBAR and PHACE syndromes. (*courtesy of Sheilagh Maguiness*, *MD*).

**FIGURE 3:**
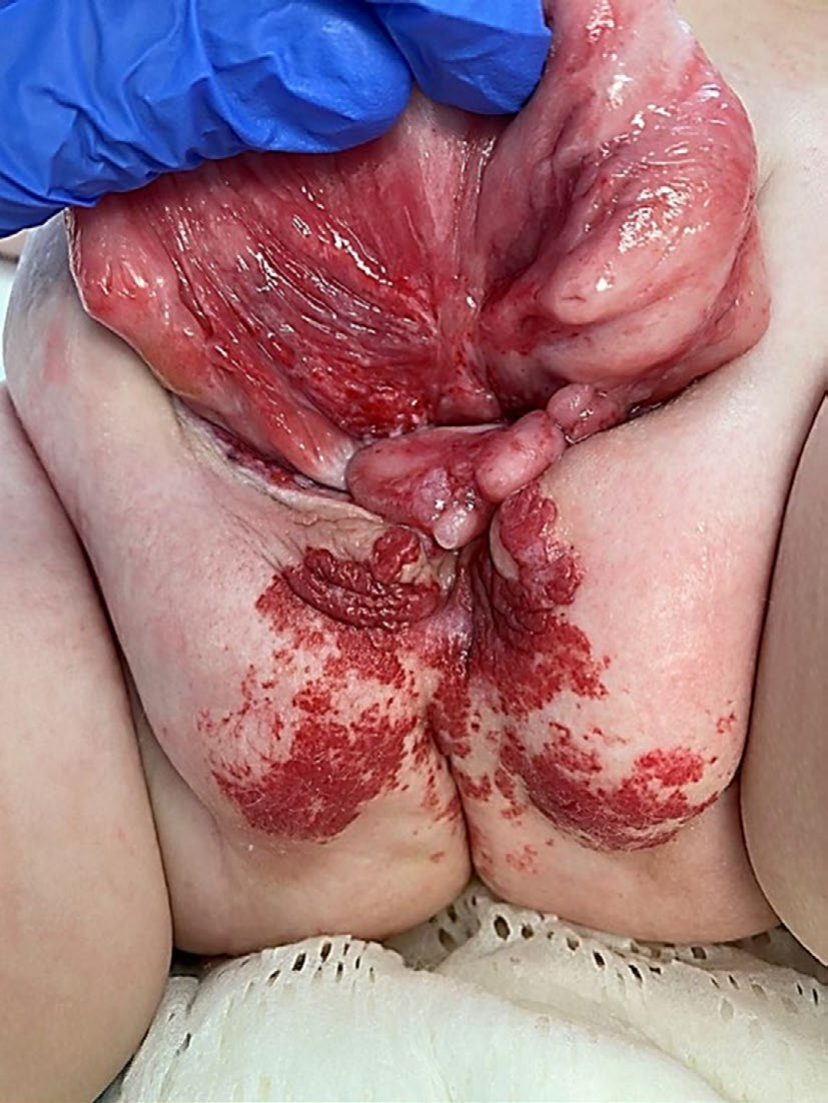
Infant with bladder exstrophy, omphalocele, and segmental infantile hemangioma of the pelvic region consistent with LUMBAR‐OEIS complex overlap. *Courtesy of Diana Bowen*, *MD and Amir Alhajjat*, *MD*.

LUMBAR shares overlapping features with two other disorders: PHACE syndrome (Posterior fossa anomalies, Hemangioma, cerebrovascular Arterial anomalies, Cardiovascular anomalies and Eye anomalies) and OEIS complex (Omphalocele, Exstrophy, Imperforate anus and Spinal anomalies), also known as cloacal exstrophy (Figure 1, Table 1) (Frieden et al. 1996; Carey et al. 1978). Both LUMBAR and PHACE are characterized by a segmental skin IH in association with other birth defects that tend to occur in regional association to the IH. In fact, LUMBAR is considered the lower body counterpart to PHACE, which affects the upper body. In contrast, the lower body congenital anomalies observed in LUMBAR very closely overlap with those observed in OEIS complex. Recent reports of infants with overlapping features of LUMBAR and PHACE (Figure 2a,b), and LUMBAR and OEIS (Figure 3), support a shared pathogenesis between these three disorders that would be unlikely to co‐occur by chance alone (Shayegan et al. 2022; Davenport et al. 2022; Barrios et al. 2024). However, although genetic and clinical risk factor studies have been performed in PHACE and OEIS, no such studies have been undertaken for LUMBAR. We thus present the first study of infant and maternal risk factors in LUMBAR syndrome.

**TABLE 1:** Compared clinical and demographic features between LUMBAR syndrome, PHACE syndrome, and OEIS complex.

Clinical and risk factors	LUMBAR syndrome	PHACE syndrome	OEIS complex
Segmental infantile hemangioma	Lumbosacral, pelvic ± leg	Head and/or neck, upper chest/arm	None
Congenital arteriopathies	Present	Present	Absent
Regional developmental anomalies	Caudal	Cephalic/cardiac	Caudal
Female predominance	63%	83%	None
Birth weight	Normal (*p* = 0.0002)	Normal	Low
Gestational age	Term (*p* < 0.0001)	Term	Preterm
Plurality	Singleton, no twins or other multiple births reported to date (*p* = 0.0605)	Typically, singleton; twins uncommon (5.7% in PHACE registry)	Multiple
Maternal age	Slightly older (*n* = 13)	Slightly older	Normal
Other maternal risk factors	No recurrent risk factors (*n* = 17)	**•** Pre‐eclampsia **•** Placenta previa	**•** Use of fertility medication **•** Assisted reproductive technology procedures **•** Pre‐pregnancy obesity **•** Periconceptional exposure to x‐rays **•** Use of progesterone or folate antagonistic medications
Family history	Not reported; one infant with a stillborn sibling found to have unilateral renal agenesis	Not reported; rare reports of siblings with isolated structural anomalies including those that are PHACE major and minor diagnostic criteria	Rare
Candidate gene analysis	Not reported	No recurrent single variants identified	No recurrent single variants identified
Difference in phenotypic severity between boys and girls	Higher incidence of anorectal, arterial, and renal anomalies in girls (not statistically significant)	Higher incidence of structural brain anomalies in boys	None

## 2 Methods

This was an IRB‐exempt, retrospective review of a database of 146 published reports used to establish diagnostic criteria for LUMBAR syndrome (Metry et al. 2024). Patients who did not meet diagnostic criteria or lacked patient‐specific data regarding gender or associated anomalies were excluded, resulting in 109 remaining individuals. We collected data on infant gender, prenatal and birth history, and congenital anomalies, maternal demographic and reproductive history, and maternal pre‐pregnancy and periconceptional exposures. Fisher's exact method was used to compare statistical significance between categorical variables.

## 3 Results (Tables 1, 2)

**TABLE 2:** A comparison of frequency of anomalies among published reports of 40 boys versus 69 girls with LUMBAR syndrome.

Sex	Male % (*N*)	Female % (*N*)	*p*‐value (Fisher's exact)
Incidence	37 (40/109)	63 (69/109)	0.0391
Urogenital anomalies	38 (15/40)	35 (24/69)	0.8492
Spinal cord malformations	80 (32/40)	74 (51/69)	0.8805
Bony anomalies	20 (8/40)	26 (18/69)	0.6528
Anorectal anomalies	25 (10/40)	45 (31/69)	0.1774
Arterial anomalies	8 (3/40)	13 (9/69)	0.5355
Renal anomalies	10 (4/40)	19 (18/69)	0.1367

There was an overall female predominance amongst LUMBAR infants with 69/109 (63%) girls and 40/109 (37%) boys. In the majority, gender was determined clinically. Six infants were reported to have “ambiguous” or “undifferentiated” genitalia but on further investigation five of the six infants had sexual underdevelopment rather than ambiguity (e.g., microphallus) thus, gender could be determined clinically. A sixth infant with LUMBAR‐OEIS complex overlap had female gender confirmed with chromosome analysis. 36/37 (97%) infants were born at term, defined as ≥ 37 weeks gestation (*p* < 0.0001). Birth weight was normal in 21/22 reports (96%), defined as ≥ 2500 g (*p* = 0.0002). 6/15 (40%) mothers were primigravids. Maternal age was reported in 13 individuals, ranging from 21 to 38 years with an average of 30.2 years and mean of 29 years (per National Vital Statistics System, average US maternal age in 2021 was 27.3 years). Pregnancy history was reported in 17 individuals and included the following single‐report complications: menotropin use at the time of conception, pre‐eclampsia in the last 3 weeks of pregnancy, and hyperemesis gravidarum throughout pregnancy. One mother had a history of hemochromatosis and vitiligo, and one pregnancy was conceived by in vitro fertilization. In 12 other individuals, maternal history was reported to be uncomplicated. Family history was negative for congenital anomalies in 13/14 reports (93%). One G2P2 mother delivered a 19‐week stillborn with unilateral renal agenesis but no other anomalies (Pelaez Mata et al. 2001). There were no reports of parental consanguinity and no reports of twins or other multiple births (*p* = 0.0605). There were no reports of other affected siblings or other family members. Chromosome analysis was performed in only three infants with normal results. Table 2 compares the incidence of LUMBAR anomalies between girls and boys. While the incidence of anorectal, arterial, and renal anomalies was higher among LUMBAR girls, this did not reach statistical significance.

## 4 Discussion

Results from our study showed that like PHACE syndrome, LUMBAR is significantly more common in full‐term, normal birth weight, singleton girls (Wan et al. 2017). In contrast, OEIS complex has been shown to be associated with preterm, low birth weight infants and with twinning (Keppler‐Noreuil et al. 2017). There have been no reports to date of LUMBAR in twins or other multiple births, though this did not reach statistical significance in our study. LUMBAR affected boys in more than one‐third of our cohort, while only about 1/6 individuals with PHACE syndrome are male (Metry et al. 2008). Comparative gender study showed a higher incidence of anorectal, arterial, and renal anomalies among LUMBAR girls, but this did not reach statistical significance. We observed no other trends in prenatal risk factors, but our numbers were small given the frequent lack of such data in published reports, the main limitation of our study.

Vascular disruption leading to in utero hypoxia could represent a pathogenic link between LUMBAR, PHACE, and OEIS. Both LUMBAR and PHACE are also characterized by regional arteriopathies, and resultant vascular disruption has been hypothesized to play a role in the hypoxic changes leading to these complications (Metry et al. 2006). LUMBAR and PHACE syndromes are both clinically recognized by the development of a characteristic segmental skin IH, which is often extensive. In LUMBAR, the IH characteristically involves the lumbosacral and/or pelvic anatomic regions, and in one‐third of patients, further extends down one or both legs (Metry et al. 2024). Regional hypoxia has been shown to play an important role in the development of a hyperproliferative vascular state triggering the development of a skin IH (Colonna et al. 2010; Drolet and Frieden 2010). Early ischemic changes and ulceration of the IH are particularly common complications in both PHACE and LUMBAR but are particularly severe in LUMBAR (Metry et al. 2024).

Furthermore, clinical overlap exists between OEIS complex, Limb–Body Wall Complex (LBWC) and Septo‐Optic Dysplasia (SOD), the pathogenesis of which is hypothesized to be due to vascular disruption (Adam et al. 2020). Genetic causes for SOD account for < 1% of cases, with the vast majority having unclear etiology. Many studies have reported an increased prevalence of antenatal drug and alcohol abuse and younger maternal age in SOD cohorts. This, in combination with the pattern of neurological abnormalities (absent septum pellucidum and optic nerve hypoplasia) found in individuals with SOD, suggests that SOD occurs secondary to a vascular disruption sequence (Lubinsky and Encha‐Razavi 2022; Lubinsky 1997; Stevens and Dobyns 2004; Webb and Dattani 2010). We recently evaluated a patient with both SOD and OEIS complex. By extension, this could lend support to vascular interruption and secondary in utero hypoxia being a contributing cause for OEIS and LUMBAR. Lastly, animal models have demonstrated that abnormal hypoxia during early embryonic development has also been associated with congenital malformations, including limb and heart defects and vertebral anomalies (Moreau et al. 2019; Sparrow et al. 2012).

No single causative gene has been identified in PHACE or OEIS; genetic studies in LUMBAR have yet to be undertaken (Siegel 2018; Vlangos et al. 2011). Reports of familial inheritance are rare or non‐existent in all three disorders. However, the possibility of an environmental insult or deficiency disrupting genetic signaling at a critical time during embryogenesis cannot be excluded. Malformations of the spinal cord, including closed neural tube defects or spina bifida occulta, are the most common extracutaneous anomalies in LUMBAR. As is true for many congenital malformations, the development of neural tube defects likely results from complex interactions between the multiple nutritional, environmental, and genetic risk factors that have been associated (Isakovic et al. 2022). Recent whole genome sequencing analysis in PHACE showed strong candidate coding and noncoding variants in genes in the RAS/PI3K pathways in individual patients with PHACE; however, no single causative gene was identified across multiple affected individuals (Partan et al. 2021). Gene ontology analyses from these studies demonstrated enrichment for vascular and mesenchymal development and morphogenesis (Colonna et al. 2010; Drolet and Frieden 2010).

**FIGURE 4:**
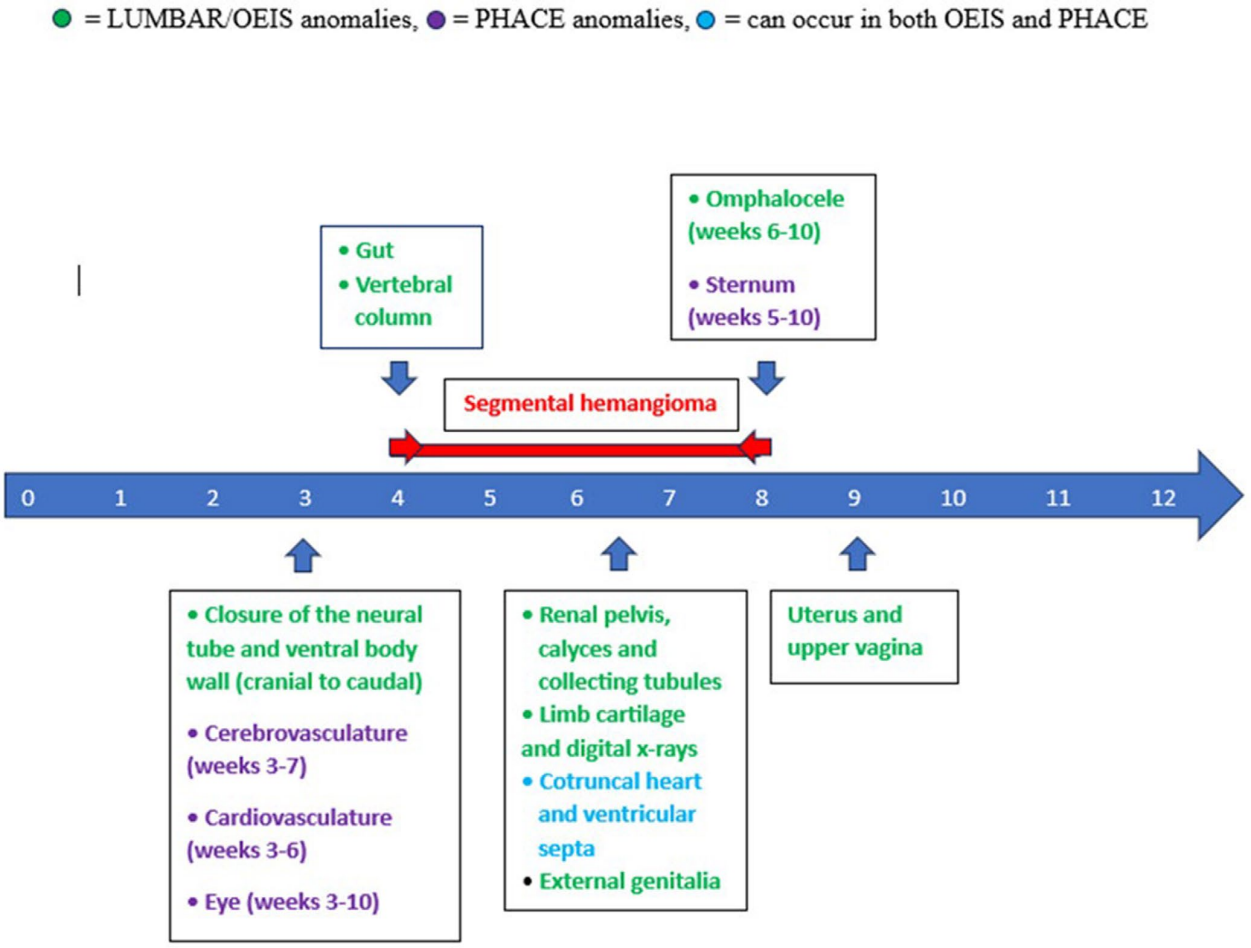
A timeline of early embryologic organ and segmental hemangioma development. Gestational weeks from birth (Week 0) to the end of the first trimester (Week 12).

The segmental skin IH and associated congenital anomalies observed in these conditions point to a developmental insult early in embryogenesis. Segmental IH is hypothesized to arise as an error in neural crest development, as early as 4 to 8 weeks gestational age, with the caudal developmental anomalies observed in LUMBAR and the cephalic anomalies typical of PHACE occurring within the first 3 to 10 gestational weeks (Figure 4) (Haggstrom et al. 2006). We hypothesize that in utero hypoxia secondary to vascular disruption, due to environmental exposure, deficiency, and/or genetic susceptibility, at a specific early embryologic timepoint could explain the association between LUMBAR, PHACE, and OEIS.

## 5 Conclusions

A shared pathogenesis between LUMBAR syndrome, PHACE syndrome, and OEIS complex is supported by recent reports of infants with LUMBAR‐PHACE and LUMBAR‐OEIS overlap. These rare disorders are unlikely to co‐occur by chance alone. Like PHACE, LUMBAR is significantly more common in full‐term, normal birth weight girls, with no statistically significant differences in phenotypic severity between girls and boys. To date, there have been no reports of LUMBAR in twins or other multiple births. While the underlying etiology is likely multifactorial, involving multiple genes and environmental risk factors, we hypothesize that in utero hypoxia secondary to vascular disruption, due to environmental and/or genetic susceptibility, at a specific early embryologic timepoint could explain the association between these three rare phenotypes. Prospective studies in LUMBAR syndrome are needed to further evaluate maternal risk factors for prenatal hypoxia, gene–environment interactions, and genetic susceptibility variants.

## Author Contributions

D.W.M. contributed to the conception and design, acquisition of data, analysis and interpretation of data, drafting of the manuscript, and revising the manuscript critically for important intellectual content. D.H.S. and K.M.K.‐N. contributed to the analysis and interpretation of data and revised the manuscript critically for important intellectual content. All authors gave final approval for the submitted manuscript and agree to be accountable for all aspects of the work in ensuring that questions related to the accuracy or integrity of any part of the work are appropriately investigated and resolved.

## Ethics Statement

This was an IRB‐exempt, retrospective review.

## Consent

Obtained for our identifiable Figure 2.

## Conflicts of Interest

The authors declare no conflicts of interest.

## Data Availability

The authors have nothing to report.
